# Sensitivity of MRI Tumor Biomarkers to VEGFR Inhibitor Therapy in an Orthotopic Mouse Glioma Model

**DOI:** 10.1371/journal.pone.0017228

**Published:** 2011-03-03

**Authors:** Christian T. Farrar, Walid S. Kamoun, Carsten D. Ley, Young R. Kim, Ciprian Catana, Seon J. Kwon, Bruce R. Rosen, Rakesh K. Jain, A. Gregory Sorensen

**Affiliations:** 1 Department of Radiology, Athinoula A. Martinos Center for Biomedical Imaging, Massachusetts General Hospital, Charlestown, Massachusetts, United States of America; 2 Edwin L. Steele Laboratory for Tumor Biology, Department of Radiation Oncology, Massachusetts General Hospital, Boston, Massachusetts, United States of America; National Cancer Institute, United States of America

## Abstract

MRI biomarkers of tumor edema, vascular permeability, blood volume, and average vessel caliber are increasingly being employed to assess the efficacy of tumor therapies. However, the dependence of these biomarkers on a number of physiological factors can compromise their sensitivity and complicate the assessment of therapeutic efficacy. Here we examine the response of these MRI tumor biomarkers to cediranib, a potent vascular endothelial growth factor receptor (VEGFR) inhibitor, in an orthotopic mouse glioma model. A significant increase in the tumor volume and relative vessel caliber index (rVCI) and a slight decrease in the water apparent diffusion coefficient (ADC) were observed for both control and cediranib treated animals. This contrasts with a clinical study that observed a significant decrease in tumor rVCI, ADC and volume with cediranib therapy. While the lack of a difference between control and cediranib treated animals in these biomarker responses might suggest that cediranib has no therapeutic benefit, cediranib treated mice had a significantly increased survival. The increased survival benefit of cediranib treated animals is consistent with the significant decrease observed for cediranib treated animals in the relative cerebral blood volume (rCBV), relative microvascular blood volume (rMBV), transverse relaxation time (T2), blood vessel permeability (K^trans^), and extravascular-extracellular space (ν_e_). The differential response of pre-clinical and clinical tumors to cediranib therapy, along with the lack of a positive response for some biomarkers, indicates the importance of evaluating the whole spectrum of different tumor biomarkers to properly assess the therapeutic response and identify and interpret the therapy-induced changes in the tumor physiology.

## Introduction

Early biomarkers of tumor response to anti-angiogenic therapy are urgently needed to enable the rapid assessment and tailoring of drug therapies. MRI biomarkers are increasingly being used to assess the efficacy of tumor therapies. In particular, MRI methods for characterizing the tumor vascular structure, including the cerebral blood volume (CBV) [1], microvascular blood volume (MBV: vascular volume pertaining only to relatively small diameter vessels) [2], [3], and vessel caliber index (VCI) [4], [5] have been developed and used to study changes in tumor vasculature with therapeutic treatment [6]–[10]. In addition, the apparent diffusion coefficient (ADC), determined from diffusion-weighted images (DWI), and the transverse relaxation time (T2) have been used as biomarkers of tumor edema [11], [12]. Finally, Dynamic Contrast Enhanced (DCE) MRI experiments [13]–[15] have been employed to assess changes in both the permeability (K^trans^) of the tumor vasculature to small Gd-based contrast agents, such as Gd-DTPA, and the volume fraction of the extra-vascular extra-cellular space (ν_e_) [16]–[18].

However, as discussed in more detail below, these MRI biomarkers typically each depend on a variety of physiological factors that may all be influenced by tumor therapy, thereby complicating the interpretation of the biomarker changes. The sensitivity of a particular biomarker may vary greatly depending on the particular tumor phenotype and what parts of the tumor physiology are being effected by a given tumor therapy. In addition, the models used to relate the biomarkers to the relevant physiology may be inadequate or poorly characterized further complicating interpretation of biomarker responses. Here we examine in detail the sensitivity of these MRI tumor biomarkers to treatment with cediranib (AstraZeneca Pharmaceuticals), a potent inhibitor of vascular endothelial growth factor receptors (VEGFR) [19]. The MRI biomarker responses are compared with both *ex vivo* histology and *in vivo* optical microscopy studies performed previously [20] in the same mouse tumor model to more directly link the biomarkers to the relevant tumor physiology and validate their sensitivity to anti-angiogenic therapy. The results reported here provide insight into which of the MRI biomarkers are most sensitive to changes in tumor morphology and predictive of tumor response to anti-angiogenic therapy.

The VCI is increasingly being used in both clinical and animal model studies as a biomarker of vessel normalization with anti-angiogenic therapy [4], [6]–[8], [21]–[23]. The VCI is defined as the ratio between the blood volume weighted for large diameter vessels (CBV, cerebral blood volume) to the blood volume weighted for small diameter vessels (MBV, microvascular blood volume) and is proportional to the average blood vessel diameter [4], [5]. Angiogenesis driven tumor growth is typically associated with increased vessel density and caliber and a disordered and tortuous vasculature structure [24]–[26]. An increased VCI with therapy has therefore typically been interpreted as a lack of a therapeutic response. However, an accurate assessment of therapeutic efficacy and interpretation of changes in the tumor vasculature is difficult to obtain from changes in the average vessel diameter alone. For example, if both the CBV and MBV decrease, suggesting a positive therapeutic response, but the MBV decreases more than the CBV then an increased VCI will be observed. Here we examine the sensitivity of the VCI to changes induced by cediranib therapy. The changes in VCI with therapy are compared with histological and intravital optical microscopy (IVM) measurements of the average tumor blood vessel diameter and CBV.

The water ADC has previously been shown to be correlated with tumor cell density [27]–[29] and has been used to monitor response to chemotherapy, where increased cell death (decreased cellular volume fraction) was associated with increased ADC [30]–[34]. However, anti-angiogenic therapies may have only anti-edema effects and the role of ADC as a sensitive biomarker of tumor edema is less well established. The ADC depends on a number of factors including the intra- and extra-cellular water diffusion coefficients and transverse relaxation times, the cellular volume fraction, and the tortuosity of the extra-cellular space [35]. Since anti-angiogenic therapies may alter a number of these parameters it is difficult to predict how the ADC will change. In addition, some anti-angiogenic therapies may have both anti-edema and anti-tumor effects that could lead to offsetting ADC responses thereby masking changes in edema. Finally, ADC may also correlate with glioma invasion [36], further complicating its interpretation over time. Given all of these possibilities, it would be helpful to explore the relationship of changes in ADC acutely after anti-VEGF therapy to changes in tumor edema. The transverse relaxation time, T2, has also been used as a biomarker of edema [11], [12]. Increased protein concentrations associated with decreased edema typically lead to decreased T2 relaxation times. However, T2 is influenced by many other factors, such as water compartmentalization and diffusion, hemorrhage, and necrosis. These factors could all lead to therapy induced T2 changes independent of changes in edema. Here we examine the sensitivity of both ADC and T2 to cediranib therapy and validate the sensitivity of these tumor edema biomarkers with *ex vivo* measurements of tumor water content determined from wet and dry tumor weights.

DCE experiments for assessing vascular permeability to low molecular weight Gd-based contrast agents, such as Gd-DTPA, require the use of an accurate arterial input function (AIF) to properly model the tracer kinetics and extract permeability parameters. However, obtaining an accurate AIF can be complicated by many factors, including partial volume and contrast agent induced T2* distortion of the measured AIF and lack of an artery in the field of view from which to measure the AIF. Here we compare the sensitivity of our DCE MRI measurements of vascular permeability changes, made using a fixed bi-exponential AIF model, with IVM measurements of changes in vascular permeability to tetramethylrhodamine-labeled bovine serum albumin (TMR-BSA).

## Materials and Methods

### Mouse Brain Tumor Model

Green fluorescent protein expressing U87 (U87-GFP) tumor cells (human glioblastoma) were grown *in vitro* (DMEM medium with 10% serum, 37 degrees, 20% oxygen, 5% CO_2_), harvested, resuspended in serum-free DMEM medium, and used for tumor implantation in athymic (*nu/nu* genotype) mice. Injections consisted of 3–5 µl of a cell suspension containing 1×10^6^ to 5×10^6^ cells/µl implanted with a 28-gauge microsyringe (10 µl, Hamilton, Reno, NV). Injections were performed with the mouse head fixed in a stereotaxic device (Small Animal Stereotaxic Instrument with Mouse Adaptor, David Kopf Instruments, Tujunga, CA). The needle tip was positioned at an angle of 55° and depth of 1.75 mm, and cells were injected slowly over 1 minute. This injection technique ensures implantation of a sufficient number of cells into the superficial mouse brain cortex. When the arising tumor reaches approximately 3×3 mm, it was harvested and divided into small fragments (∼0.5×0.5 mm) for implantation in the animals used for the MRI experiments. The tumor fragments were implanted into the mice by dissecting the skin from a small area of the skull, slightly anterior to the bregma and lateral to the midline. A small portion of tumor tissue was implanted with a 30-gauge needle in the exposed brain. Mice were imaged 10–14 days following tumor implantation (day 0), when tumors typically reached a diameter of 2–3 mm, and again 2–3 days later following cediranib treatment, which consisted of 3–6 mg cediranib/kg bodyweight/day. Control animals were treated with Tween. The Massachusetts General Hospital Subcommittee on Research Animal Care approved all experiments (SRAC protocol # 2004N000050).

### Intravital Optical Microscopy (IVM)

Green fluorescent protein (GFP) expressing U87-GFP tumors were implanted in athymic mice, as described above, with previously implanted cranial windows [37], [38]. When tumors reached a diameter of 1.8–3.5 mm, animals were anaesthetized and 3–6 locations per animal were imaged using a multi-photon laser-scanning microscope. To visualize the vessels, 150 µl of tetramethylrhodamine labeled dextran (MW 2 million, 10 mg/ml) was injected intravenously. Stacks of 250 µm depth with 5 µm Z-steps were acquired, and a virtual vascular cast was generated in 3D by custom image analysis software [39]. Length-weighted average vessel diameter was calculated based on the virtual cast. A relative Cerebral Blood Volume (rCBV_IVM_) was calculated from the ratio of the tumor to contralateral cortex microvessel density (MVD) determined from the IVM data.

For vascular permeability measurements, tetramethylrhodamine labeled BSA (TMR-BSA) was injected intravenously and the fluorescence signal in a single optical section containing a vessel of interest was monitored, as described previously [25]. The permeability *P* of a vessel of radius *r* was then calculated from Equation 1, given below.
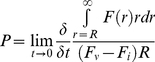
(1)Here *F*_ν_ is the fluorescence intensity from the plasma in the vessel and *F*_i_ is the fluorescence intensity immediately outside the vessel. The integral of *F*(*r*), the fluorescence intensity in the extravascular space, was evaluated numerically along a line perpendicular to the flow axis of the vessel. This derivation assumes that the relationship between fluorescence signal and local concentration of fluorophore is uniform across the line of interest and there is no influx from adjoining vessels.

### Magnetic Resonance Imaging

All experiments were performed on a 9.4 Tesla magnet (Magnex Scientific Ltd, Oxford, UK) equipped with a 60 mm inner diameter gradient coil (Resonance Research, Billerica, MA) and interfaced with a Bruker MRI console (Bruker Biospin, Billerica, MA). The gradient coil has a maximum strength of 1500 mT/m and a rise time of 100 µs. Images were acquired using either a home built surface coil or a home built mouse head bird-cage coil. Mice were positioned on a custom made mouse cradle and anesthetized with 1.5% isoflurane in 50/50 O_2_/medical air mixture with total flow rate of 1200 ml/min. Contrast agent injections were performed using an intravenous tail vein catheter.

For assessing the response of the U87 mouse brain tumor to cediranib it was necessary to split the mice into two groups to minimize the scanner time, since it was found that repeated long exposure to anesthesia resulted in animal death. The imaging protocol for the first group of animals was as follows: localizer sequence, T2-weighted RARE sequence, multi-echo spin-echo sequence (T2 map), multi-echo gradient-echo sequence (T2* map), injection of 50 µl of SPION (8 mg Fe/ml or ∼16 mg Fe/kg bodyweight), multi-echo spin-echo sequence (T2 map), multi-echo gradient-echo sequence (T2* map). The imaging protocol for the second group of animals consisted of: localizer sequence, T2-weighted RARE sequence, multi-echo spin-echo sequence (T2 map), spin-echo diffusion weighted sequence (DWI), DCE sequence (100 repetitions) with injection of 50 µl of 100 mM Gd-DTPA. Total scan time in each case was just under 1 hour.

### T2-weighted RARE MRI

T2-weighted Rapid Acquisition with Refocused Echoes (RARE) images were acquired to assess the tumor volume. The acquisition parameters were: TE = 10, RARE factor = 16, TR = 3000 ms, NA = 4, 11 image slices, 0.5 mm slice thickness, 150 µm in-plane resolution. Tumor volume was determined from the T2 hyperintense tumor region of the brain.

### Steady-State Susceptibility Contrast (SSC) MRI

T2 and T2* maps were generated from multi-echo spin-echo and multi-echo gradient-echo images, respectively, using a custom written MATLAB program for voxel-wise fitting of the T2 or T2* relaxation times. Multi-echo spin-echo image acquisition parameters were: TE = 10 ms, 10 echoes with 10 ms increment, TR = 2.5 s, 2 averages, FOV = 1.92 cm, matrix = 128×128 (in-plane resolution 150 µm), slice thickness = 0.5 mm, 11 image slices. Multi-echo gradient-echo image acquisition parameters were: TE = 2.5 ms, 8 echoes with 2.5 ms increment, TR = 1.0 s, 4 averages, FOV = 1.92 cm, matrix = 128×128 (in-plane resolution = 150 µm), slice thickness = 0.5 mm, 11 image slices. Images were acquired both before and after injection of SPION (16 mg Fe/kg bodyweight, r2 = 40 mM^−1^ s^−1^). The relative Cerebral Blood Volume (rCBV) weighted for large diameter blood vessels (ΔR2*) and relative microvascular blood volume (rMBV) weighted for small diameter blood vessels (ΔR2) were determined from the difference between the post- and pre-SPION R2* (1/T2*) and R2 (1/T2) maps respectively. A tumor relative VCI (rVCI) was calculated using Equation 2 where the tumor VCI was normalized to the normal contralateral cortex VCI. The value of the Apparent Diffusion Coefficient (ADC) was taken from DWI measurements (see below).
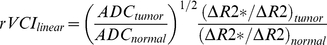
(2)


### Diffusion Weighted Imaging (DWI):

Spin-echo diffusion weighted images were acquired with 3 different b-values: 0, 756, 1506 s/mm^2^. Spin-echo acquisition parameters were: TE = 13.8 ms, TR = 3 s, FOV = 1.92 cm, matrix = 128×128 (in-plane resolution = 150 µm), 0.5 mm slice thickness, 11 image slices. The apparent diffusion coefficient (ADC) maps were generated using an in-house written MATLAB program for fitting the natural log of the signal intensity as a function of b-value.

### Dynamic Contrast Enhanced (DCE) Imaging:

The DCE sequence consisted of a T1-weighted gradient-echo sequence with TE = 2.5 ms, TR = 50 ms, Flip Angle = 35°, FOV = 1.92 cm, matrix = 96×96 (in-plane resolution = 200 µm), 0.5 mm slice thickness, 1 image slice, 70–100 repetitions, temporal resolution = 4.8 s. 50–100 µl of 100 mM Gd-DTPA (0.2–0.4 mmoles/kg) was injected approximately 30 s after commencement of the DCE imaging sequence. The signal intensity in the tumor ROI was analyzed using an in-house written MATLAB program, which models the tumor signal enhancement using the two-compartment model of Tofts *et al* [13]–[15], to extract the volume fraction of the extra-vascular extra-cellular (EES) space (*ν_e_*), the volume transfer constant between the plasma and EES (*K^trans^*), and the rate constant between the EES and the blood plasma (*k_ep_*). Briefly, the time dependence of the tumor signal intensity is fit to equation 3.
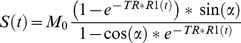
(3)where *R1(t)* is the longitudinal relaxation rate, *a* is the flip angle, and *TR* is the repetition time. *R1(t)* depends on the contrast agent relaxivity (*r_1_*), the pre-contrast longitudinal relaxation rate (*R1(0)*), and the tissue concentration of the contrast agent tracer (*C_t_(t)*) as described by equation 4.
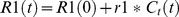
(4)In turn, *C_t_(t)* is derived from the arterial input function (AIF), *C_p_(t)*, as described by equation 5.
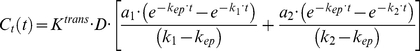
(5)

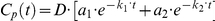
The AIF is modeled as a bi-exponential function with parameters *a_1_* and *k_1_* describing the fast equilibration between the plasma and extracellular space, *a_2_* and *k_2_* describing the clearance of contrast agent by the kidneys, and *D* is the contrast agent dose (mmoles Gd/kg bodyweight) administered by intravenous injection [13]. We have used the AIF parameters determined empirically by McGrath *et al* [40].

### Histology

Tumor-bearing mice were perfusion fixed by infusion of 4% paraformaldehyde through the left ventricle. For immunofluorescence analysis, mouse brains were post-fixed for 1 hour in 4% formaldehyde in PBS followed by incubation in 30% sucrose in PBS overnight at +4°C and subsequent mounting in freezing media (OCT, Tissue-Tek). Brains were sectioned every 20 µm and incubated for 2–4 hours at room temperature with anti-CD31 antibody (2.5 µg/ml, clone 2H8, Millipore Chemicon International) in 0.2% Triton-X100 and 5% normal horse serum (NHS) in PBS. After several washes in PBS, tissue sections were incubated for 1 hour at room temperature with 1∶400 dilutions of Cy5-conjugated anti-armenian hamster antibody in 0.2% Triton-X100 and 5% NHS in PBS. After several washes in PBS, tissues were post-fixed in formaldehyde and mounted with DAPI containing mounting media (Vectashield, VectorLabs) for confocal microscopy. Quantification of the stained area was performed using an in-house segmentation algorithm (coded using MATLAB, Mathworks).

### Ex Vivo Tumor Water Content Analysis

Anesthetized mice were euthanized by cervical dislocation and the brains were collected. Brains were dissected into several compartments: Tumor, ipsilateral hemisphere, contralateral hemisphere and midbrain. Tissues were weighed immediately and dried in a vacuum for up to 2 weeks. Weights were collected throughout the drying period until the final dry weight was established. Water content was calculated as following:
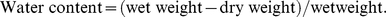



### Statistical Analysis

Comparisons of changes in the MRI biomarkers between the cediranib and control groups were performed using analysis of variance (ANOVA) calculations and statistical significance was defined by a p-value of <0.05.

## Results

**Figure 1:**
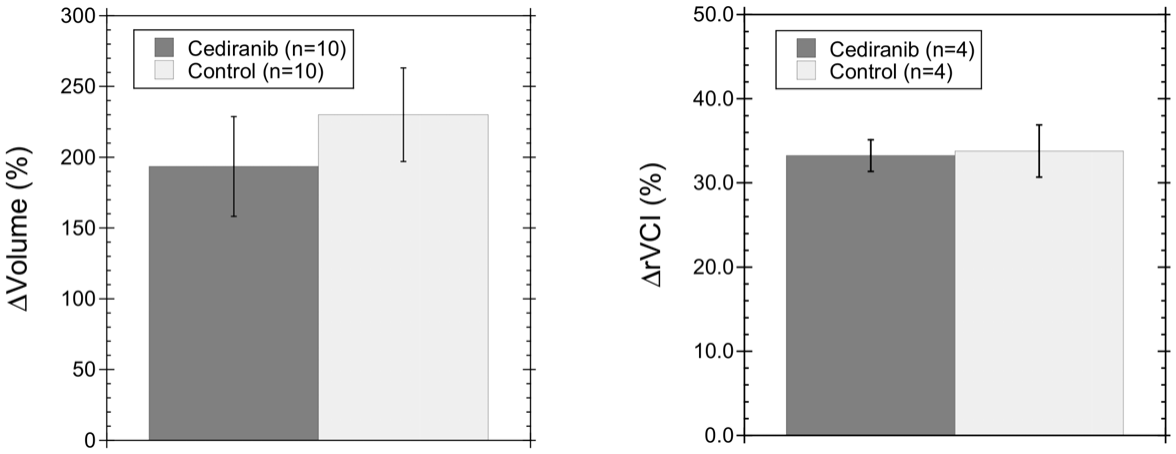
Cediranib therapy does not affect tumor growth or vessel caliber. (left) Percent change after 2–3 days of treatment in the (left) tumor volume and (right) rVCI for cediranib and control treated animals. A significant increase over time in tumor volume and rVCI was observed for both cediranib and control animals with no statistically significant difference between treatment groups.

Tumor volume was determined from the T2-weighted hyperintense region on day 0 and day 2–3 following commencement of treatment. An increased tumor volume was observed over time for both cediranib (194±35%) and control (230±33%) animals with no statistically significant difference between groups (Figure 1, left). This is in agreement with previous IVM measurements of tumor volume, which observed no difference in tumor growth rate between cediranib and control animals [20].

Similarly, an increased rVCI was observed on day 2–3 for both cediranib (33.2±1.9%) and control (33.8±3.1%) animals with no significant difference between treatment groups (Figure 1, right). This result is in good agreement with previous histology measures of the average tumor blood vessel diameter [20], which saw no differences between control (9.4±0.3 µm) and cediranib (9.5±0.4 µm) treated animals in the average vessel diameter of the tumor core after 2 days of treatment.

**Figure 2:**
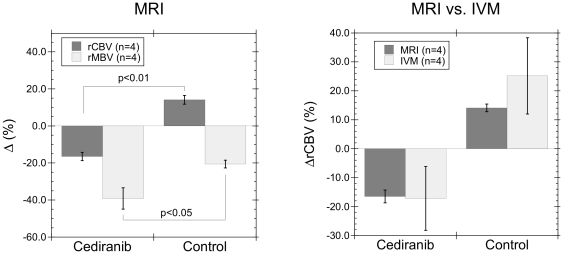
Cediranib therapy decreases tumor blood volume. (left) After 2–3 days of treatment, a statistically significant (p<0.01) difference in the rCBV (±SEM) was observed between cediranib and control groups with a 16.5±2.2% decrease for cediranib treated animals and a 14.1±2.3% increase for control animals. In contrast, after 2–3 days of treatment, the rMBV decreased for both cediranib (−39.2±5.8%) and control (−20.6±2.1%) groups. (right) The changes over time in rCBV measured by MRI and IVM are in excellent agreement, with a large decrease in rCBV for cediranib treated animals and a large increase in rCBV for control animals observed by both imaging modalities.

While the rVCI increased equally for cediranib and control groups, the increase occurred for different reasons. As shown in Figure 2 (left panel), cediranib treatment lead to a decrease in both rCBV (−16.5±2.2%) and rMBV (−39.2±5.8%). The larger drop in rMBV compared to rCBV lead to the increased rVCI observed in Figure 1. In contrast, the control group observed an *increase* in rCBV (+14.1±2.3%) and a decrease in rMBV (−20.6±2.1%), again resulting an increased rVCI. A statistically significant difference between control and cediranib groups was observed in both the change over time of the rCBV (p<0.01) and of the rMBV (p<0.05). As demonstrated in Figure 2 (right panel), there were no statistically significant differences between MRI and IVM measurements of the change over time in rCBV for either control or cediranib treated animals.

**Figure 3:**
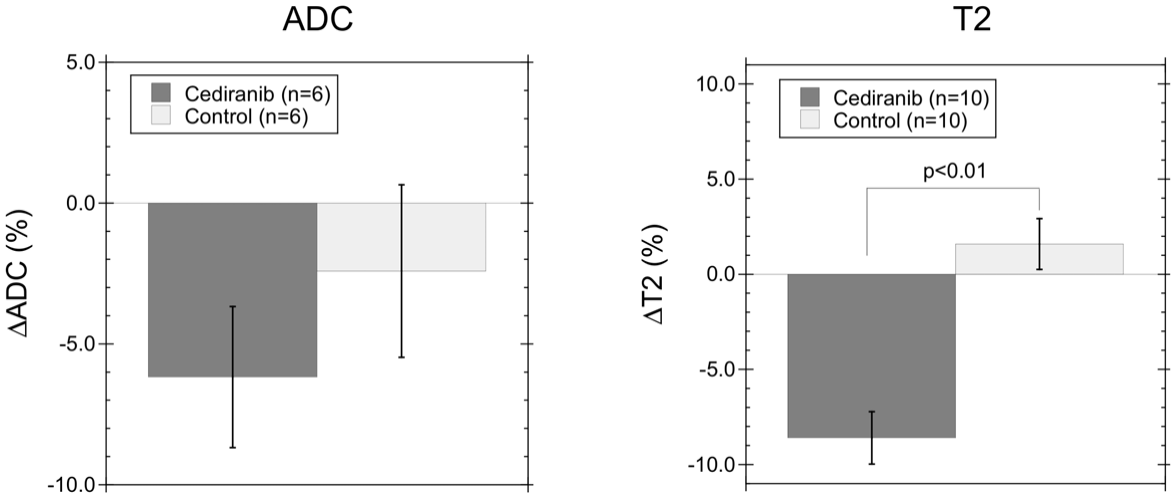
Cediranib therapy decreases tumor T2, but does not affect ADC. (left) While a significant decrease in ADC was observed for cediranib treated mice (p = 0.01) after 2–3 days of treatment, no statistically significant difference was observed between cediranib and control groups for the change over time of ADC. (right) In contrast, the decreased T2 observed after 2–3 days of treatment for cediranib animals was significantly (p<0.01) different from the increased T2 observed for control animals.

While there was a statistically significant (p = 0.01) decrease in ADC (−6.2±2.5%) with cediranib treatment, the ADC also decreased slightly for control animals (−2.4±3.1%) with no statistically significant difference between cediranib and control groups (Figure 3, left). In contrast, a significant (p<0.01) difference in the change over time of T2 (ΔT2) was observed between control (1.6±1.3%) and cediranib (−8.6±1.4%) treated animals (Figure 3, right). The decreased T2 for cediranib treated animals is consistent with the decreased water content measured *ex vivo* from the wet and dry tumor weights on day 2 of treatment, where a 6.3±1.9% decrease in tumor water content was observed between cediranib and control animals [20]. This is in good agreement with the 8.5±0.2% difference in T2 observed on day 2 by MRI for control (T2 = 52.8 ms) and cediranib (T2 = 48.3 ms) treated animals.

**Figure 4:**
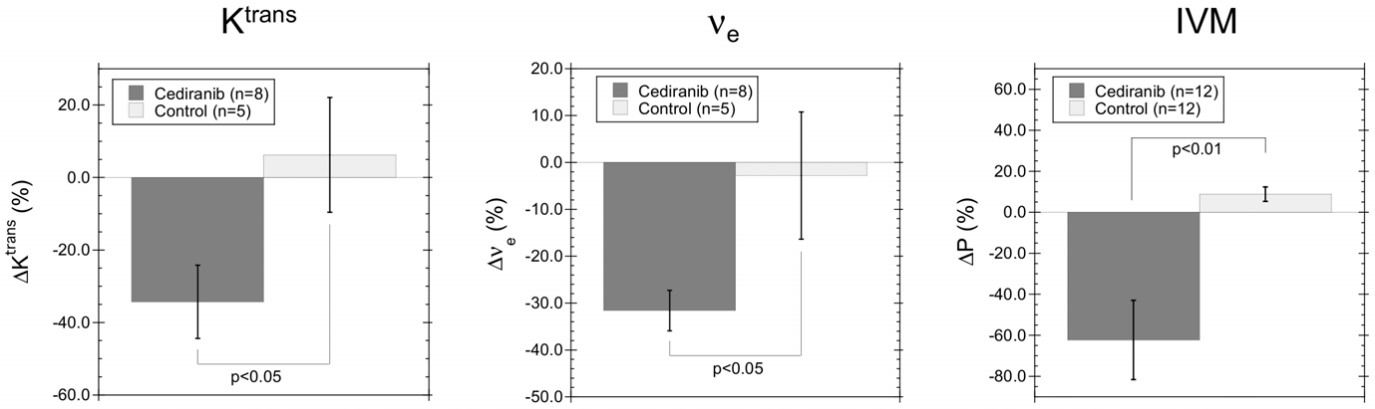
Cediranib therapy decreases tumor vascular permeability and extravascular-extracellular space. A significant decrease in both K^trans^ (left) and ν_e_ (middle) was observed after 2–3 days of treatment with cediranib. A statistically significant decreased vascular permeability to BSA (DP) was also observed by IVM after 2 days of cediranib treatment (right), consistent with the decreased permeability over time observed by MRI (K^trans^).

A statistically significant difference between cediranib and control groups was observed in both the change over time of K^trans^ (p = 0.04) and ν_e_ (p = 0.03). A 34.3±10.1% decrease in K^trans^ (±SEM) was observed over time for cediranib treated animals, while a 6.3±15.8% increase in K^trans^ was observed for control animals (Figure 4, left). Similarly, changes over time in ν_e_ (±SEM) of −31.6±4.3% and −2.8±13.6% were observed for cediranib and control groups, respectively (Figure 4, middle). The changes in vascular permeability to Gd-DTPA (K^trans^) observed by MRI are in good agreement with the changes in permeability to tetramethylrhodamine-labeled bovine serum albumen (TMR-BSA) observed previously [20] by IVM (Figure 4, right), where a decreased permeability (ΔP = −62.3±19.2%) for the cediranib group and an increased permeability (ΔP = 8.8±3.5%) for the control group were observed on day 2.

## Discussion

While many MRI biomarkers of anti-angiogenic therapy response have been proposed, the sensitivity of these biomarkers to anti-VEGF therapy has not been examined in detail [41]. In particular, these biomarkers typically each depend on a number of physiological factors that when altered by tumor therapy may lead to opposing effects on the biomarker response, thereby minimizing the biomarker sensitivity to therapy. A recent clinical study of 30 recurrent glioblastoma patients treated with a single dose of cediranib (AstraZeneca Pharmaceuticals), a potent VEGF receptor-targeted kinase inhibitor, did observe a strong correlation between changes in the MRI biomarkers K^trans^ and microvascular blood volume (MBV) and the duration of overall and/or progression-free survival [42]. In addition, recent IVM and MRI studies in a U87 mouse brain tumor model demonstrated that cediranib significantly prolongs survival despite persistent tumor growth, where the survival benefit was primarily attributed to decreased vascular permeability and reduction of edema [20]. Here we extend these studies by examining the response of multiple MRI tumor biomarkers, including CBV, MBV, VCI, K^trans^, ν_e_, T2, and ADC, to cediranib therapy and comparing them to previously reported [20] histology and IVM measurements of the tumor physiology, including tumor water content, average blood vessel diameter, blood volume, and vascular permeability. These studies therefore help to identify the biomarkers that are most sensitive to changes induced by anti-angiogenic therapy and to more directly link the biomarker responses to changes in the relevant tumor physiology.

Recent studies have shown that the vascular models used to derive the relationship between the average blood vessel diameter and the MRI measured CBV (ΔR2*) and MBV (ΔR2) may be inadequate for modeling the very abnormal tumor vasculature [43], [44]. The vasculature is typically modeled as a random uniformly distributed collection of perfect cylinders [45], [46]. Not only may this vascular model be inadequate for tumors, but also it is unclear how anti-angiogenic tumor therapies that normalize the tumor vasculature will affect the appropriateness of such a fixed vascular model. Here we find that the increased rVCI observed for both control and cediranib treated animals (Figure 1) is consistent with histology measurements of the average vessel diameter [20], which observed no difference in vessel diameter between control and cediranib treated animals after 2 days of treatment. This suggests that despite the simplistic static vascular model used, the VCI does accurately reflect changes in the average blood vessel diameter.

Cediranib treated mice have been shown to have a significantly increased survival rate compared to controls [20]. Evaluation of the rVCI alone therefore might mistakenly suggest that cediranib has no therapeutic benefit as the VCI increased significantly for both cediranib and control animals. However, analysis of changes in the rCBV and rMBV indicates that the increased rVCI for control and cediranib groups occurred for quite different reasons (Figure 2). While the rCBV and rMBV both decreased significantly for cediranib treated animals, the rMBV decreased more than the rCBV resulting in an increased rVCI. In contrast, for control animals the rCBV *increased* while the rMBV decreased, again resulting in an increased rVCI. The larger decrease over time in the MBV compared to the CBV observed for cediranib treated animals suggests that cediranib is preferentially pruning smaller caliber, less mature tumor blood vessels and has a smaller, but still significant, effect on the larger blood vessels. In contrast, the *increased* CBV and decreased MBV observed over time for control mice would be consistent with an increasingly avascular tumor with vessel regression in the tumor core and fewer, but larger, blood vessels. Such progression to an avascular phenotype for the core of large tumors is not uncommon and has, for example, been observed previously in a rat glioma model [47].

The change in rCBV measured by MRI is in excellent agreement with that measured by IVM (Figure 2). In addition, the strong response of the rCBV and rMBV to cediranib therapy is consistent with a previous clinical study where cediranib treatment lead to a significant decrease in both rCBV and rMBV [7]. While the clinical study did observe a transient decrease in rVCI that was not observed in our mouse brain tumor model, this might simply reflect somewhat different relative responses of the rMBV and rCBV in the clinical subjects, where a greater decrease is observed for the rCBV than rMBV. Finally, in contrast to the VCI, the decreased rMBV measured after only one treatment in the clinical study was strongly correlated with the duration of overall and/or progression-free survival [42]. These results therefore suggest that the CBV and MBV may be better gauges of therapeutic response than the VCI.

Changes in the T2-weighted signal intensity of tumors are frequently taken as evidence of changes in tumor edema [11]. Here we quantified tumor T2 relaxation times and compared the changes in T2 with changes in tumor water content measured *ex vivo*. A significant difference between cediranib and control groups is observed in the T2 response (Figure 3). In particular, cediranib treated animals had an 8.5±0.2% lower T2 on day 2 than control animals, which is in good agreement with the 6.3±1.9% decreased tumor water content measured *ex vivo* after 2 days of cediranib therapy [20]. This suggests that T2 is a sensitive and quantitative biomarker of changes in tumor edema. However, care must be taken when analyzing tumors with regions of necrosis and hemorrhage. While the U87 tumor model studied here displayed no sign of necrosis, hypointense regions consistent with hemorrhage were evident in some cases, particularly along the periphery of the tumor. It is therefore important to define tumor regions-of-interest that do not contain hemorrhage as this will result in significantly decreased T2 values. In addition, therapies that induce large changes in water compartmentalization (i.e. due to necrosis and/or changes in the extravascular-extracellular space) and diffusion could also complicate interpretation of T2 changes. Changes in water compartmentalization and water diffusion would, however, be reflected in the ADC. For the U87 tumor model studied here, only very small changes in ADC were observed with therapy (Figure 3).

The ADC is also frequently used as a biomarker of tumor edema. While the ADC did decrease significantly over time for cediranib treated animals, the ADC also decreased slightly for control animals leading to no significant differences in the ADC response to treatment between cediranib and control groups (Figure 3). In contrast, tumor edema determined from tumor T2 and from *ex vivo* wet-dry tumor weights [20] showed a significant decrease in tumor edema over time for cediranib treated animals compared to controls. The ADC depends on a large number of factors including the intra- and extra-cellular water diffusion coefficients and transverse relaxation times, the cellular volume fraction, and the tortuosity of the extra-cellular space [35]. The significant decrease in ADC for cediranib treated animals likely resulted from a combination of decreased edema and decreased extravascular-extracellular space (increased cellular volume fraction), observed by DCE MRI (Figure 4). In contrast, the slight decrease in ADC for control animals likely resulted from an increase in tumor edema, which would lead to an increased ADC, being offset by the decrease in the tumor extravascular-extracellular space (Figure 4), which leads to a decreased ADC. The decreased extravascular-extracellular space for control animals may be a result of the increased tumor blood volume (Figure 2) and an increased tumor cell volume induced by increased intra-cellular water content. This illustrates that offsetting responses in different parts of the tumor physiology can compromise the sensitivity of the ADC to changes in tumor edema. Thus, for this tumor model, T2 is a more sensitive gauge of changes in tumor edema than ADC. This is in agreement with a previous clinical study, which found T2 to be more sensitive and “useful” than ADC for differentiating contrast-enhancing tumor and immediate edema regions [11], though it conflicts with clinical experience with cediranib where early changes were seen on ADC before they were seen on T2-weighted images [7]. The dependence of the MRI biomarkers on a number of physiological factors points to the need to consider the biomarker changes in relation to one another to properly interpret the therapy induced changes. Only by considering the ADC, T2, and ν_e_ responses together, for example, can insight be obtained into the likely changes in tumor physiology that are occurring for cediranib and control groups. In general, the sensitivity of a particular MRI biomarker may vary greatly depending on which physiological factors are being altered most by a given therapy for a particular tumor.

Finally, DCE experiments using low molecular weight Gd-based contrast agents, such as Gd-DTPA, are routinely performed for assessing changes in vascular permeability (K^trans^) in response to anti-angiogenic therapy. However, accurate measurement of K^trans^ requires the use of an accurate arterial input function (AIF) to properly model the tracer kinetics. Obtaining an accurate AIF is complicated by many factors, including partial volume and contrast agent induced T2* distortion of the measured AIF and lack of an artery in the field of view from which to measure the AIF. In particular, for mouse brain images obtained with a surface RF coil, no arteries are typically visible from which to measure an AIF. Using a reference tissue, such as scalp tissue, to calibrate the tumor DCE curves can in principle allow the tracer kinetic parameters to be determined without the need for a direct AIF measurement [48]. However, in practice using a reference tissue calibration is complicated by B_1_ field inhomogeneities associated with surface coils and B_0_ field inhomogeneity present at air-tissue interfaces, such as the scalp. Recent studies suggest that given the large potential errors in measurement of the AIF, it is better to use an assumed, fixed AIF model for all subjects [49]. The validity of such an approach, however, remains unclear. The AIF is sensitive to blood flow velocity (linked to body weight), blood pressure (dependent on anesthesia dose and body temperature), and the amount of contrast agent injected (variable due to manual injection method typically used for animal studies), all parameters that are difficult to assess with great accuracy and likely vary from subject to subject. While in theory an accurate measure of AIF could help reduce all of these sources of subject-to-subject variability, in practice measurement of the AIF may itself add uncertainty, rather than reduce it, and thus these uncertainties in many of the kinetic tracer model input parameters may lead to large uncertainties in the assessment of the vessel permeability parameters K^trans^ and ν_e_. A previous study of a subcutaneous flank tumor model in rats, where an AIF was directly measured and compared with a variety of AIF models, suggests that errors introduced by using a fixed bi-exponential AIF model are less than 5% [40]. However, others have suggested that the use of a fixed AIF model can lead to large systematic errors in the determination of permeability parameters [13], [50]. The agreement between permeability parameters extracted using experimentally measured and fixed models is likely to be highly variable depending on the tumor model and reproducibility of the experimental techniques used (i.e. anesthesia, contrast agent injection technique, etc.). The decreased permeability to Gd-DTPA induced by cediranib observed in this study (Figure 4), as quantified by K^trans^, is consistent with the decreased permeability to BSA observed previously by IVM [20]. The IVM measurement of permeability involves a much simpler and more straightforward analysis of the fluorescent signal intensity in the vascular and extravascular spaces, with no complicated kinetic modeling or need for an accurate AIF. The good agreement between DCE and IVM permeability measurements therefore suggests that the simple, fixed bi-exponential AIF model can be adequate for assessing changes in vascular permeability. The strong response of K^trans^ to cediranib therapy is again in agreement with a previous clinical study, which saw a significant decrease in K^trans^ for cediranib treated subjects [7] that was correlated with the duration of overall and/or progression-free survival [42].

In summary, the MRI biomarkers T2, K^trans^, ν_e_, rCBV, and rMBV all decreased significantly in response to cediranib therapy. The decreased T2 was correlated with decreased tumor water content measured *ex vivo*, indicating that T2 is a sensitive biomarker of tumor edema. In contrast, the ADC was not sensitive to changes in tumor water content in this tumor model. The decreased K^trans^ was consistent with IVM measurements of vascular permeability. The changes in rCBV were in good agreement with IVM measures of tumor blood volume changes. In addition, the biomarker responses observed here are consistent with a previous clinical study that observed a strong decrease in K^trans^, rCBV and rMBV with cediranib treatment [7]. These findings indicate that T2, K^trans^, rCBV, and rMBV are sensitive biomarkers of tumor response to anti-angiogenic therapy in this tumor model. The fact that the VCI and ADC were not sensitive to cediranib therapy in our mouse brain tumor model, in contrast to the clinical cediranib study [7], suggests that the sensitivity of a particular MRI biomarker varies depending on which physiological factors are being altered most by a given therapy for a particular tumor. It also indicates the importance of measuring the whole spectrum of MRI tumor biomarkers and examining their changes in relation to one another in order to properly assess the therapeutic response and identify and interpret the therapy induced changes in the tumor physiology.
